# Clinical significance of activated Wnt/β-catenin signaling in apoptosis inhibition of oral cancer

**DOI:** 10.1515/biol-2021-0104

**Published:** 2021-09-28

**Authors:** Yufeng Wang, Zheng Cao, Fengjia Liu, Yuejian Ou

**Affiliations:** Department of Stomatology, Huzhou Central Hospital, Affiliated Central Hospital of Huzhou University, No. 1558 Third Ring North Road, Huzhou, Zhejiang, 313000, China

**Keywords:** apoptosis, β-catenin, cell proliferation, oral cancer, tumorigenesis

## Abstract

Wnt/β‐catenin signaling is an evolutionarily conserved pathway and plays a crucial role in regulating cancer cell proliferation and tumorigenesis. However, the molecular mechanism behind the Wnt/β‐catenin signaling-mediated carcinogenesis and apoptosis resistance in oral squamous cell carcinoma is not well characterized so far. In the present study, we have investigated the effect of β‐catenin depletion of the perversely activated Wnt/β-catenin signaling pathway on apoptosis resistance and tumorigenesis of the human OSCC cell line SCC-55. RT-PCR and western blot analysis demonstrated that the Wnt/β-catenin signaling pathway and its downstream targets such as DKK1 and AXIN2 are aberrantly activated in SCC-55 cells. Furthermore, upon silencing (RNA interference) of β‐catenin in SCC-55, cells became more sensitive toward the chemotherapeutic drugs and thus resulted in apoptotic cell death. Meanwhile, flow cytometry analysis confirmed the enhanced apoptosis and activation of caspases in β‐catenin RNAi cells. Besides ensuing β-catenin–siRNA transfection, the cell proliferation and cancer colony generating efficiencies are significantly impeded compared to the non-transfected cells. Furthermore, the tumorigenicity was inhibited by the downregulation of *OCT-4* in β‐catenin-silenced SCC-55 cells. Altogether, Wnt/β‐catenin signaling could potentially target anti-cancer drugs to induce apoptosis and achieve a better clinical outcome.

## 1 Introduction

Oral squamous cell carcinoma (OSCC) is the most common type of oral cancer that originates in the oral mucosa or dysplasia in oral mucosa or dysplasia, signifying the sixth most common cancer in the world [1,2]. OSCC is associated with an aggressive type of differentiated tumors and extensive metastasis to the lymph node region even at an early stage. In the past few years, upon diagnosis, the average survival rate of OSCC patients (less than 50%) at the metastasis stage is about 5 years [3] and thus indicates oral cancer has significant health implications worldwide. Conventional treatment methods for OSCC patients include surgery, chemotherapy, radiotherapy, and phytotherapy in recent times [3,4]. *Oral carcinogenesis* is a multifaceted process that comprises both genetic and molecular modifications that result in defected DNA repair mechanisms, modulated signaling pathways, deregulated cell cycle, and limited apoptosis.

The irrevocable genetic and epigenetic modifications in oral cancers lead to distorted protein expression and functions, such as Notch1, EGFR, cyclin, p53, pRB, and Wnt/β-catenin pathway factors [5,6,7,8]. Among them, the Wnt/β-catenin signaling has been proposed as an essential pathway, plays a crucial role in cell survival and tumorigenesis [9,10]. Aberrant activation of Wnt/β-catenin signaling has been reported in different types of human cancers [11,12,13,14], characterized by β-catenin nuclear translocation and overexpression of Wnt ligands. As a result, accelerated cancer growth and tumorigenesis occur by activating downstream targets of Wnt/β-catenin signaling [15].

Previous reports have demonstrated that Wnt/β-catenin signaling pathways are significantly upregulated in oral cancer cells and malignant oral lesions [13,14,15,16]. However, the research data regarding the transcriptional regulation of Wnt/β-catenin signaling components and their molecular mechanism in cell survival, proliferation, apoptosis resistance, and tumorigenic potential are still lacking. Therefore, meticulous research for molecular and signaling pathways involved in oral carcinogenesis is imperative to develop novel anti-cancer drugs with potential targets. Considering this fact, in the present study, we have performed an RNA interference (RNAi) approach for β-catenin to unravel the Wnt/β-catenin signaling targets and its regulation involved in apoptosis resistance, cell proliferation, and tumorigenesis of oral squamous cell carcinoma cell line SCC-55.

## 2 Materials and methods

### 2.1 Cell line and cell culture

The specifications of the SCC-55 cell line used in this study are as follows: site – oral squamous cell carcinoma; region – mandibular, recurrence type of tumor; tumor grade – III. As a control, we have used healthy (non-cancerous) oral squamous epithelial cells obtained in the mandibular region, and no lymphatic nodes are affected. Control cells are named HS-129 and, hereafter, throughout this article, the HS-129 cell line is referred to as “control.” Both SC-55 and HS-129 cell lines are maintained in our Huzhou Central Hospital cell culture depository. These cell lines were cultured in DMEM (Dulbecco’s modified Eagle’s medium; Thermofisher Scientific) containing fetal bovine serum (10%) and antibiotics such as penicillin and streptomycin. The cell culturing conditions/parameters, passaging, and cryopreservation were performed as described previously [17].

### 2.2 siRNA transfection

SCC-55 cells were grown on six well-plates for 24 h in DMEM with 10% FBS at 37°C with a supply of 5% CO_2_. Study groups were assigned as (a) SCC-55 (non-transfected which acts as control); (b) SCC55 + scramble RNAi; and (c) SCC-55 + β-catenin RNAi. Cells at the logarithmic growth phase were subjected to RNAi transfection, mediated by transfection reagent 6 μL of Lipofectamine^®^ 2000 (Invitrogen). The transfection procedure was performed exactly as described in the manufacturer’s protocol. The targeting siRNA for the β-catenin gene (CATGUGUTGGUAAGCUCUA) and scramble RNAi sequence (AUGCUGATCAGUGUCGATU) were used as described previously [14], and they were purchased from Gene Pharma, Co., Ltd, Shanghai.

### 2.3 Reverse transcription (RT)-polymerase chain reaction (PCR)

Complementary DNA was prepared from the extracted total RNA using the Fermentas Reverse Transcriptase kit. Subsequently, 1 μL of reverse transcription reaction was subjected to qPCR (Bio-Rad-iCycler) with a total volume of 30 μL reaction with Biorad IQ supermix (Syber Green). The primer sequences for *AXIN2* and *DKK1* used in this study were as described previously [18,19]. The primers sequences for β-catenin, *OCT-4, BMI-1*, and *GAPDH* were described previously [20,21]. OCT-4: F- GCAATTTGCCAAGCTCCTGAA and R-GCAGATGGTCGTTTGGCTGA; GAPDH: F-ATGTCGTGGAGTCTACTGGC and R-TGACCTTGCCCACAGCCTTG; β-catenin: F-GCTACTCAAGCTGATTTGATGGA and R-GGTAGTGGCACCAGAATGGATT. PCR parameters include 35 cycles 95°C for 30 s (denaturation), 58°C for 40 s (annealing), 72°C for 30 s (extension) and 72°C for 7 min (final extension). 1% agarose gel electrophoresis with ethidium bromide (EtBr) staining was used to visualize the amplicons. The mRNA band intensity was measured using ImageJ software, and the relative mRNA expression level was adjusted with the GAPDH gene. The change in the expression level (in the fold) was also measured by using the 2-ΔΔcT method.

### 2.4 Luciferase assay

Cells were grown for 24 h, subjected to transfection with a reaction mix containing TOPFLASH/FOPFLASH of 75 ng and pCMV-RL (20 ng), mediated by Lipofectamine^®^ 2000. Cell lysates were prepared from 24 h post-transfected cells, and the luciferase activity was determined according to the manufacturer’s protocol (Promega Dual-Luciferase Reporter Assay System).

### 2.5 Western blot

Total cellular proteins were separated by 10% sodium dodecyl sulfate (SDS)-polyacrylamide gel electrophoresis (PAGE). Subsequently, proteins were transferred to nitrocellulose membranes and blocked with a 5% skim milk solution in TBST. Then, they were incubated with the primary antibodies such as cytochrome c (Cell Signaling, 1:500); caspase 3 (Cell Signaling, 1:1,000); caspases 9 (1:1,000); BCL-2 (1:2,000) and GAPDH (1:5,000; Cell signaling); Axin2 (1:500); DKK1 (1:2,000) and β-catenin (1:1,000) from Thermofishers. The HRP conjugated secondary antibody, either mouse or rabbit conjugated, was purchased from cell signaling (1:5,000). The protein signal was determined with the ECL chemiluminescence kit (Biorad ECL).

### 2.6 Flow cytometry analysis

Propidium iodide staining (Sigma-Aldrich Kit) was performed in 48 h post-transfected siRNA cells. After overnight incubation at 37°C in dark conditions, cells were fixed in 70% ice-cold methanol and the apoptosis rate was evaluated by flow cytometry. The apoptosis percentage was measured by the mean intensity of the fluorescence signal emitted by dead cells. The mean values represented in the quantification graph were obtained from three individual experiments.

### 2.7 *In vitro* cell proliferation assay

Cells (10^6^) were seeded in 96 well plates, cultured, and the proliferation rate at OD 450 nm was measured from day 1 to day 7. Before each measurement, CCK-8 solution (10 μL) was added to the culture and incubated for 3 h. Thus, the growth curve represents the average OD values of three independent experiments.

### 2.8 Chemoresistance assay

Cells were seeded in 96 well plates and grown for 24 h. Then they were treated with 5-fluorouracil (5-FU;10 μg/mL) and cisplatin (20 μmol/L). After 48 h, 10 μL of CCK-8 solution was added to wells, incubated for 3 h, and then chemoresistance assay was performed. The resistance was evaluated precisely using the formula as described previously [22]: rate of cell resistance (%) = (experimental group OD_450_ value/control group OD_450_ value) × 100.

### 2.9 Soft agar assay

The six-well plates were made of two types of agar layers: bottom layer – 6% agar in DMEM with 10% FBS; top layer – 0.3% agar in DMEM with 10% FBS. Upon solidification, approximately 1 × 10^4^ cells were seeded in the top layer and incubated for 2–3 weeks at 37°C. Subsequently, crystal violet staining was performed to visualize and count the colonies.

### 2.10 Statistical analysis

The statistical software package was used to perform statistical analysis. The values represented in the error bars are ± standard deviation (SD). In addition, for statistical comparisons between two groups, a one-way analysis of variance (ANOVA) was performed.

## 3 Results

### 3.1 Aberrant activation of Wnt/β-catenin signaling in oral squamous cell carcinoma

**Figure 1:**
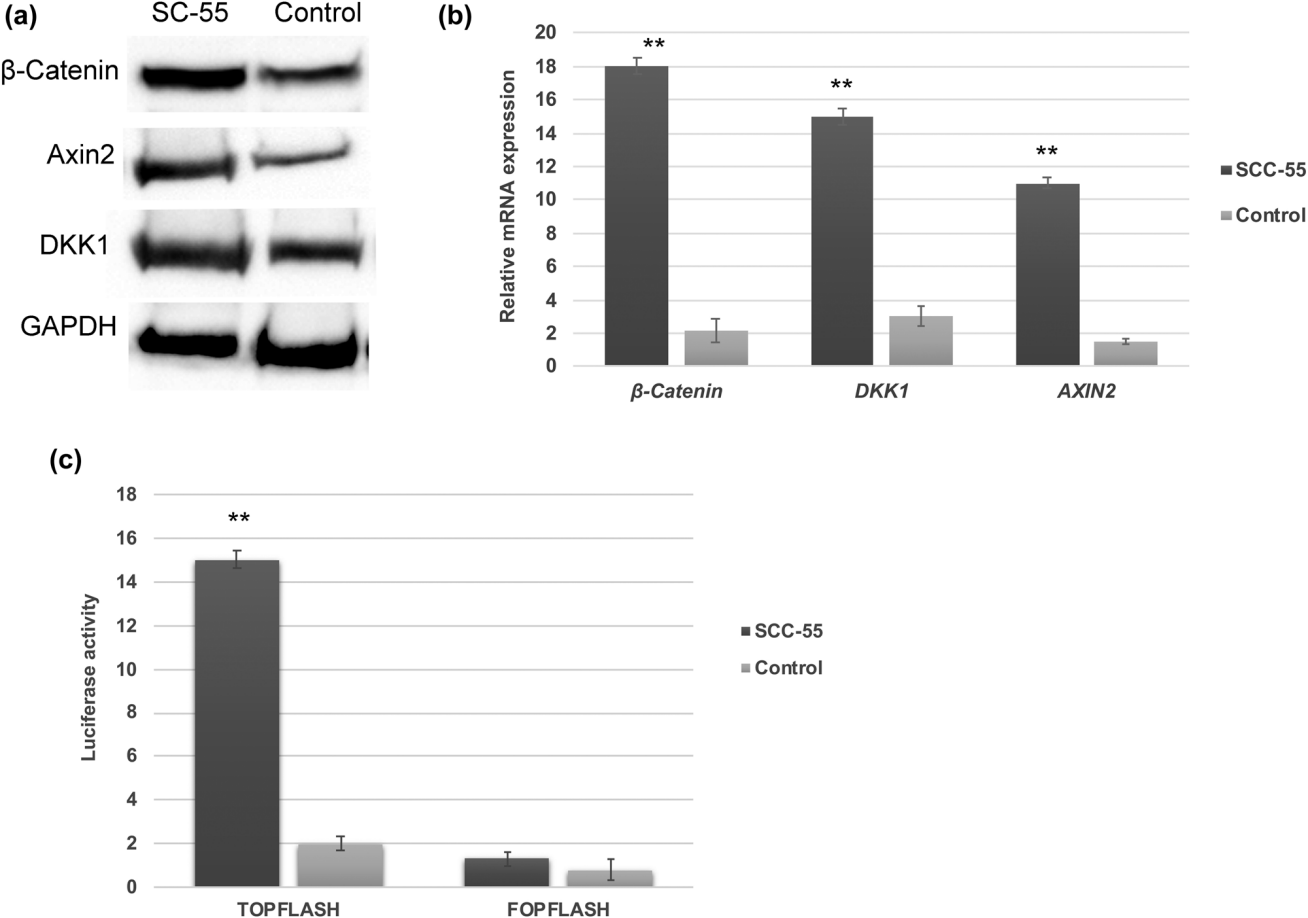
Aberrant activation of Wnt/β-catenin signaling in OSCC. Western blot (a) and RT-PCR analysis (b) showing that Wnt/β-catenin and its downstream targets are activated and upregulated in OSCC SCC-55 cells. (c) TOPFLASH luciferase activity showing the elevated transcriptional level of Wnt/β-catenin in SCC-55 cells. The values in the error bar are± standard deviation (SD); ** *p* < 0.01.

To investigate whether Wnt/β-catenin signaling is activated in oral squamous cell carcinoma (OSCC) SCC-55 cells, we have examined the expression pattern of Wnt/β-catenin genes and its downstream targets. As a result, by western blot analysis, the protein expression level of β-catenin and the downstream targets such as DKK1 and Axin2 are highly upregulated in oral cancer SCC-55 cells compared to control cells (Figure 1a). Analogously, our RT-PCR analysis revealed that the transcriptional upregulation of genes such as *β-catenin* (>7-fold), *DKK1* (>5-fold), and *AXIN2* (>7-fold) than control cells (Figure 1b). Conclusively, we have performed another confirmatory assay, such as the TOP FLASH luciferase reporter assay. In this assay, the activation of Wnt/β-catenin is directly correlated to the activation of the TOPFLASH reporter gene. Moreover, the FOPFLASH reporter gene acts as a negative control as it contains mutated β-catenin. Furthermore, luciferase reporter assay data displayed significantly enhanced transcriptional activation of Wnt/β-catenin activity in SCC-55 cells than control cells (Figure 1c). Therefore, these data suggest that Wnt/β-catenin activity is aberrantly upregulated in SCC-55 cells.

### 3.2 β-catenin depletion influences apoptosis in SCC-55 cells

**Figure 2:**
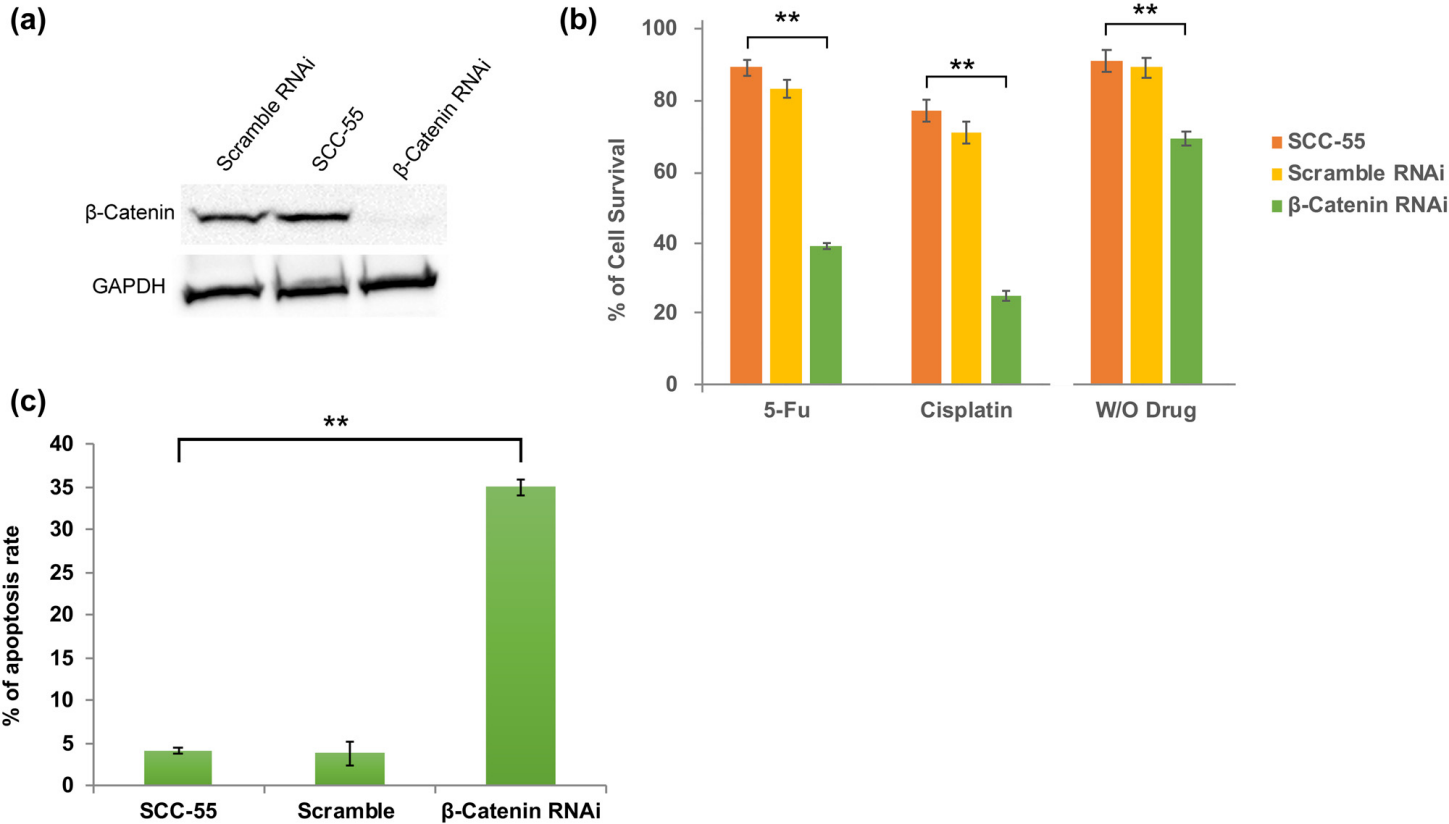
β-catenin knockdown induces apoptosis in SCC-55 cells. (a) Western blot data showing decreased β-catenin expression after β-catenin siRNA transfection in SCC-55 cells. (b) Chemoresistance assay showing that β-catenin RNAi cells viability are significantly reduced. (c) Flow cytometry assessment confirms the enhanced rate of apoptosis in β-catenin RNAi SCC-55 cells when compared to controls. The values in the error bar are ± standard deviation (SD); **, *p* < 0.01.

As Wnt/β-catenin signaling is highly activated in SCC-55 cells, we attempted to knock out β-catenin by RNA interference approach. As a result, our western blot analysis demonstrated that β-catenin expression was dramatically reduced upon β-catenin–siRNA transfection in SCC-55 cells when compared to scramble RNAi and non-transfected (control) SCC-55 cells (Figure 2a). Cancer cells are highly resistant to apoptosis, and therefore, they developed resistance to chemotherapy. Hence, the β-catenin depleted cells are subjected to the evaluation of chemotherapy resistance and apoptosis induction. As a result, we found that SCC-55-β-catenin RNAi cells became more sensitive to DNA-binding drugs such as 5-FU and cisplatin. As a result, the survival rate of β-catenin RNAi cells significantly declined and thus indicated apoptosis (Figure 2b). In addition, the evaluation of apoptosis by flow cytometry revealed that the enhanced rate of apoptosis in β-catenin-depleted SCC-55 rather than scramble RNAi and non-transfected SC-55 cells (Figure 2c). Collectively, these findings suggest that the downregulation of β-catenin was able to induce apoptosis effectively in oral cancer cells, and therefore, cancer cell survival was compromised.

### 3.3 β-catenin downregulation impedes cell proliferation and tumorigenesis

**Figure 3:**
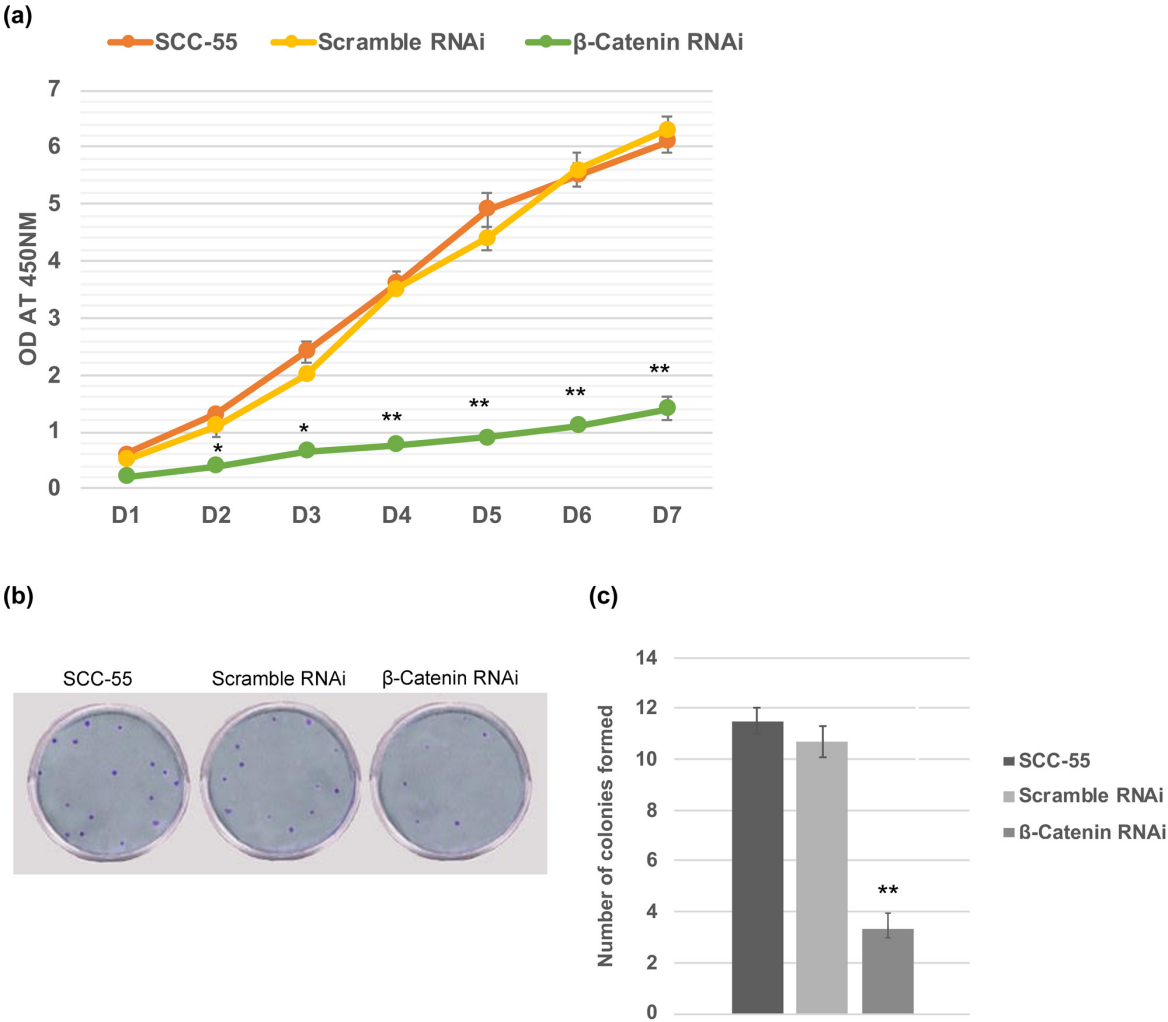
β-catenin knockdown impedes cell proliferation and inhibits tumorigenicity. (a) *In vitro* cell proliferation assay displaying that the growth rate of SCC-55 cells was compromised upon silencing of β-catenin. (b) The number of colonies formed in the soft agar assay was dramatically less upon siRNA of β-catenin transfection in SCC-55 cells. The values in the error bar are ± standard deviation (SD). * *p* < 0.05; ** *p* < 0.01.

Next, we have examined whether the β-catenin downregulation has a deleterious effect on the tumorigenic potential of oral cancer cells. *In vitro* proliferation assay demonstrated that the proliferation rate of β-catenin-depleted SCC-55 cells was significantly deteriorated from day 1 to day 7 than the non-transfected and negative control ones (Figure 3a). Also, the colony formation efficiency of β-catenin RNAi cells was reduced (<3-fold times), as they regenerated significantly fewer colonies on soft-agar assay (Figure 3b). Hence, these data indicate that β-catenin–siRNA was able to limit cell survival and self-renewal properties of oral cancer cells, which may be governed by the interaction of stemness genes.

### 3.4 Inactivation of caspases and activation of Oct-4 by Wnt/β-catenin signaling

**Figure 4:**
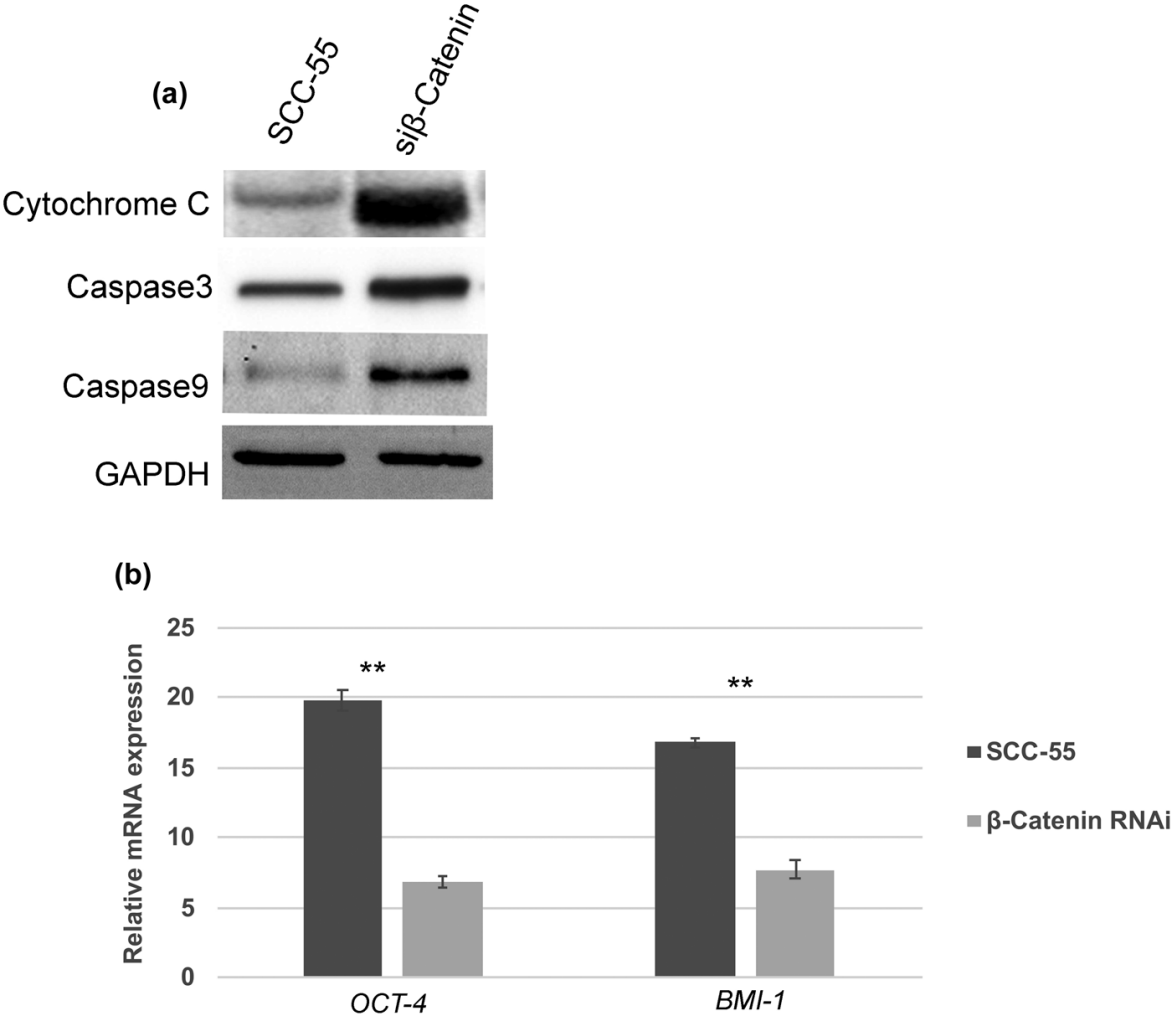
Activation of intrinsic apoptotic pathway and downregulation of OCT-4 in β-catenin RNAi cells. (a) Western blot analysis showing activation and enhanced expression of cytochrome c, caspase 3, and caspase 9 in β-catenin RNAi SCC-55 cells. (b) RT-PCR data showing relative mRNA expression of OCT-4 was significantly downregulated in β-catenin RNAi cells compared to the controls. The values in the error bar are ± standard deviation (SD); ** *p* < 0.01.

It has been well demonstrated that cytochrome c release is crucial for the onset of mitochondrial-mediated intrinsic apoptosis through the activation of caspases. Consequently, the protein expression pattern of cytochrome c, caspases 3 and 9 were evaluated by western blot analysis, and they were all found to be highly upregulated in β-catenin–siRNA cells (Figure 4a). Concurrently, the relative mRNA expression of the *OCT-4* gene was accelerated in β-catenin–siRNA cells (Figure 4b), compared to non-transfected SCC-55 cells. Altogether, these data suggest that β-catenin downregulation might promote caspases activation, leading to apoptosis in β-catenin–siRNA cells. On the other hand, β-catenin–siRNA caused decreased tumorigenic potential, which could be due to transcriptional downregulation of *OCT-4*.

## 4 Discussion

In recent times, tumor initiation, progression, and invasion have become complex mechanisms that involved many cell adhesion molecules and intracellular signaling pathways for cell migration and invasion [23,24]. Mainly, Wnt/β-catenin signaling has gained more attention as it plays a crucial role in regulating cell proliferation, self-renewal, and differentiation of cancer cells and embryonic stem cells [25]. According to pathological studies, human neck and squamous cell carcinoma (HNSCC) has been associated with hyperactivation of Wnt/β-catenin signaling, contributing to HNSCC invasion [26,27,28]. In the present study, we have found abnormal activation of Wnt/β-catenin signaling in OSCC SCC-55 cells, whose protein and relative mRNA expression are significantly upregulated in SCC-55 cells.

β-catenin is an essential stimulation center for Wnt signaling pathways; cytoplasmic multifunctional protein can interact with E-cadherin adhesion molecule in a calcium-dependent manner [29,31]. Overexpression of β-catenin has been reported in different cancers, including breast, skin, blood, head, and neck [11,12,13], and they are associated with high-risk tumorigenesis and invasion [14]. Thus, it has been suggested that two different roles might be performed by the aberrantly activated Wnt/β-catenin signaling in cancer cells: (a) promote tumorigenesis and invasion and (b) suppress cell detachment-mediated apoptosis [28]. Keeping this in mind, we investigated whether β-catenin depletion influences apoptosis and tumorigenic potential of SCC-55 cells.

In the present article, we have used siRNA that could specifically target β-catenin and suppress its expression. Our RT-PCR and western blot data confirmed the significantly downregulated levels of β-catenin, and thus, the siRNA used could effectively inhibit the Wnt signaling pathway. Interestingly, our flow cytometry analysis demonstrated that apoptosis was significantly initiated in β-catenin-depleted SCC-55 cells. As a result, these cells became more susceptible when treated with DNA targeting drugs such as 5-FU and cisplatin. The precise molecular mechanism of apoptosis induction in SCC-55 cells upon β-catenin gene depletion is not known. However, we found elevated caspase-3 and caspase-9 in β-catenin RNAi cells, indicating that PI3/AKT signaling might be downregulated, promoting mitochondria-mediated intrinsic apoptotic pathways [30,31,32,33]. Similar to our findings, studies in the colon cancer cell line demonstrated that knocking of β-catenin significantly increased the rate of apoptosis through the activation of caspase-3 and limited the tumor invasion process in SW480 cells [14].

The present study has also demonstrated that the cell proliferation rate slowed down dramatically, and the number of colonies formed in the soft agar assay was significantly compromised when the β-catenin gene expression was downregulated. By considering these data, it can be suggested that the silencing of β-catenin may directly disrupt the expression of genes involved in the regulation of the cell cycle, cell proliferation, and self-renewal of oral cancer cells. Accordingly, our RT-PCR analysis revealed that *OCT-4* gene transcription was significantly downregulated in SCC-55 cells upon β-catenin silencing. Elevated expression of *OCT-4* has been identified in several cancers, and it acts as a critical regulator of cancer invasion and colony formation [34,35,36]. Furthermore, knockdown of *OCT-4* results in decreased tumor invasion and colony formation efficiency in lung cancer [35].

On the other hand, it has been reported that the therapeutic drug resistance of liver cancer cells is mediated by overexpression of *OCT-4* [35,36]. Therefore, our results suggest here that Wnt/β-catenin signaling aberrant activation and traits in oral squamous cell carcinoma SCC-55 cells might occur through the activation of *OCT-4*. However, further research is required for elucidating the β-catenin-mediated anti-apoptotic mechanism, and its downstream effectors would undoubtedly provide more insights for precisely disrupting the cascade events of Wnt signaling pathways to kill the cancer cells.
